# Effect of different soybean meal type on ileal digestibility of amino acid in weaning pigs

**DOI:** 10.1186/s40781-015-0041-9

**Published:** 2015-03-17

**Authors:** Dong Hyuk Kim, Pil Seung Heo, Jae Cheol Jang, Song Shan Jin, Jin Su Hong, Yoo Yong Kim

**Affiliations:** College of Agriculture and Life Science, Seoul National University, 151-921 Seoul, South Korea; School of Agricultural Biotechnology, Seoul National University, 1 Gwanak-ro, Gwanak-gu, Seoul, 151-921 Republic of Korea

**Keywords:** Fermented soybean meal, Probiotics, UV-sterilization, Weaning pigs, Ileal amino acid digestibility

## Abstract

An experiment was conducted to evaluate apparent (AID) and standardized (SID) ileal digestibilities of crude protein (CP) and amino acids (AA) with 6 soybean products in weaning pigs. A total of 14 weaning barrows with an initial body weight of 6.54 ± 0.34 kg were fitted with T-cannula at the distal ileum and allotted to 7 diets containing various soybean products. The soybean products used in the experiment were conventional soybean meal (CSBM), SBM fermented by *Aspergillus oryzae* GB-107 (FSBMA), SBM fermented by *Bacillus subtilis* PP6 (FSBMB), UV sterilized SBM fermented by *Bacillus subtilis* PP6 (UVFSBMB), SBM containing *Bacillus subtilis* PP6 (PSBM), and soy protein concentrate (SPC). Six corn-based diets were used and each of soybean products was added. All diets contained 5.0 g/kg of chromic oxide as an indigestible indicator and an N-free diet was used to measure basal endogenous losses of CP and AAs. Ileal CP digestibility did not differ by different soybean products. However, SIDs of Ile, Phe and Val were improved in pigs fed the FSBMB, UVFSBMB and SPC diets and the pigs fed the FSBMA diet showed higher SIDs of Phe and Val compared with those fed the CSBM diet (P < 0.05). The FSBMB diet had higher SIDs in most AAs compared with the FSBMA diet (P < 0.05), and higher SIDs of Lys, Ala, Pro, Ser, and Tyr compared with PSBM diet (P < 0.05). However, there was no response of UV-sterilization on the FSBMB in the SIDs of AAs. These results suggest that SIDs of AAs could be improved by the supplementation of fermented soybean products in the diet for weaning pigs but fermentation with *Bacillus subtilis* is more efficient in improving ileal AA digestibility than that with *Aspergillus oryzae*. Furthermore, probiotics supplementation in the CSBM and UV-sterilization of the FSBMB had no effects on chemical composition and ileal AA digestibility.

## Background

Soybean meal (SBM) is widely used in swine diets as a protein source, but contains various anti-nutritional factors (ANFs) such as trypsin inhibitor, oligosaccharides, antigenic factors and lectin. Anti-nutritional factors limit nutrient availability in weaning pigs [1,2]. Thus, many efforts to improve the nutrient availability of SBM have been made over the past decade.

Recently, fermented SBM (FSBM) inoculated with probiotic bacteria was introduced and evaluated in the diet of weaning pigs [3]. Fermentation of SBM by probiotic bacteria such as *Aspergillus oryzae* or *Bacillus subtilis* could degrade ANFs, produce small size peptide compared to conventional SBM (CSBM), and modify amino acid (AA) profiles by microbial AA synthesis and breakdown, resulting in improvement of the AA digestibility [2-4]. Previous studies demonstrated that FSBM supplementation improved protein digestibility in weaning pigs, which resulted from trypsin inhibitor elimination and high activities of protease and trypsin in the small intestine [2,5]. However, there is limited information about ileal AA digestibility of FSBM and other soybean products, and the potential differences of digestibility between FSBMs fermented by different bacterial species. Thus, a comparative study is clearly needed for efficient use of these soybean products in the diet of weaning pigs.

Therefore, the objective of this study is to evaluate apparent ileal digestibility (AID) and standardized ileal digestibility (SID) of crude protein (CP) and AA in different types of soybean products.

## Methods

The protocol for the present experiment was approved by the Institutional Animal Care and Use Committee of Seoul National University, Seoul, Republic of Korea.

### Experimental design and diets

A total of 14 weaning barrows {[Yorkshire × Landrace] × Duroc; average body weight (BW) of 6.54 ± 0.34 kg} were equipped with simple T-cannula at distal ileum followed by Stein et al. [6] at weaning (24 d of age). Pigs were allotted to one of 7 diets in a completely randomized design (CRD) and the collection period was spilt into 2 separate groups (Group 1, n = 14; Group 2, n = 14). Dietary treatments were divided by different soybean products as followings: 1) CSBM: conventional SBM, 2) FSBMA: SBM fermented by *Aspergillus oryzae* GB-107, 3) FSBMB: SBM fermented by *Bacillus subtilis* PP6, 4) UVFSBMB: UV-sterilized FSBMB, 5) PSBM: SBM containing 0.1% of probiotics (*Bacillus subtilis* PP6 strain, 1.0 × 105 CFU/g), 6) SPC: soy protein concentrate, and 7) N-free diet: as used to calculate endogenous losses of pigs. All products of FSBM were obtained from feed company (CJ Cheiljedang Co. Ltd, Incheon, Korea). The PSBM was produced by 0.1% addition of *Bacillus subtilis* PP6 grown on Luria broth in the CSBM as a probiotics. Corn based diets with 130 g/kg of barley were used with each assigned amount of different soybean products. Each experimental diet contained 13.7 MJ of ME/kg, 210.0 g/kg CP, 12.4 g/kg lysine and 3.7 g/kg methionine, and other nutrients were met or exceeded NRC [7] requirement estimates (Tables 1 and 2). To make practical standard for swine industry, soy oil, monocalcium phosphate and limestone were added for energy and Ca/P balance. Since the CP contents of each soybean product were not equal, synthetic lysine and methionine were added to the experimental diets with different levels of the soybean products (369.0, 298.1, 303.6, 303.6, 369.5, and 232.0 g/kg; CSBM, FSBMA, FSBMB, UVFSBMB, PSBM, and SPC, respectively). The N-free diet consisted of corn starch as a main ingredient and contained same levels of vitamins and minerals with other diets. Chromic oxide was added to all experimental diets at 5.0 g/kg as an indicator.

**Table 1:** Composition of experimental diets, as-fed basis^1^

**Ingredients, g/kg**	**CSBM**	**FSBMA**	**FSBMB**	**UVFSBMB**	**PSBM**	**SPC**	**N-free**
Corn	360.2	450.8	441.3	441.3	357.5	520.4	-
CSBM	369.0	-	-	-	369.5	-	-
FSBMA	-	298.1	-	-	-	-	-
FSBMB	-	-	303.6	-	-	-	-
UVFSBMB	-	-	-	303.6	-	-	-
SPC	-	-	-	-	-	232.0	-
Cornstarch	-	-	-	-	-	-	703.4
Dextrose	-	-	-	-	-	-	150.0
Lactose	80.0	80.0	80.0	80.0	80.0	80.0	80.0
Soy oil	26.1	10.4	12.4	12.4	27.2	5.1	22.0
Barely	130.0	130.0	130.0	130.0	130.0	130.0	-
Monocalcium phosphate	16.8	11.4	11.4	11.4	16.8	11.5	26.4
Limestone	6.2	7.3	8.5	8.5	6.3	8.5	7.0
L-lysine · HCl	-	0.4	1.1	1.1	-	0.8	-
DL-methionine	0.5	0.4	0.5	0.5	0.5	0.5	-
Vitamin premix^2^	1.2	1.2	1.2	1.2	1.2	1.2	1.2
Mineral premix^3^	1.0	1.0	1.0	1.0	1.0	1.0	1.0
Salt	2.0	2.0	2.0	2.0	2.0	2.0	2.0
Zinc oxide	1.0	1.0	1.0	1.0	1.0	1.0	1.0
Chromic oxide	5.0	5.0	5.0	5.0	5.0	5.0	5.0
Choline chloride	1.0	1.0	1.0	1.0	1.0	1.0	1.0
Probiotics^4^	-	-	-	-	1.0	-	-
Total	1,000.0	1,000.0	1,000.0	1,000.0	1,000.0	1,000.0	1,000.0

^1^CSBM: conventional soybean meal, FSBMA: soybean meal fermented by *Aspergillus oryzae* GB-107, FSBMB: soybean meal fermented by *Bacillus subtilis* PP6, UVFSBMB: UV-sterilized soybean meal fermented by *Bacillus subtilis* PP6, PSBM: probiotics-supplemented soybean meal, and SPC: soy protein concentrate.

^2^Provided per kg of diet: vitamin A, 16,000 IU; vitamin D_3_, 3,200 IU; vitamin E, 35 IU; vitamin K_3_, 5 mg; vitamin B_2_, 6 mg; vitamin B_12_, 20 ug; Capantothenate, 16 mg; Biotin, 128 ug; Niacin, 32 mg.

^3^Provided per kg of diet: Fe, 281 mg; Cu, 288 mg; Mn, 49 mg; Se, 0.3 mg; I, 0.3 mg.

^4^ *Bacillus subtilis PP6* (1.0 × 10^5^ CFU/g) grown on Luria broth.

**Table 2:** **C**hemical composition of analyzed experimental diets

**Treatments** ^**1**^							
**Items**	**CSBM**	**FSBMA**	**FSBMB**	**UVFSBMB**	**PSBM**	**SPC**	**N-free**
**Chemical composition, g/kg**							
Dry matter	911.4	902.1	921.2	915.0	913.5	918.5	908.2
Crude protein	218.0	203.5	212.8	203.6	222.1	203.2	4.2
**Amino acid composition**							
Dietary indispensable amino acids, g/kg							
Arg	13.6	12.0	12.2	11.5	14.1	12.9	0.1
His	5.0	4.6	4.8	4.6	5.2	4.8	0.2
Ile	8.5	7.6	8.0	7.5	8.6	8.0	0.2
Leu	16.8	15.6	16.2	15.5	17.2	16.5	0.3
Lys	11.4	11.0	11.8	10.3	11.7	12.7	0.2
Met	3.6	3.2	3.9	3.8	4.2	3.8	0.1
Phe	9.5	8.7	9.3	8.8	9.7	9.1	0.2
Thr	8.1	7.6	7.6	7.2	8.6	7.7	0.1
Val	9.0	8.5	8.7	8.4	9.1	8.7	0.3
Dietary dispensable amino acids, g/kg							
Ala	9.4	9.1	9.2	8.9	9.9	9.4	0.1
Asp	21.0	19.0	19.5	18.6	21.8	19.6	0.1
Cys	3.2	3.3	3.3	3.5	3.4	3.1	0.1
Glu	38.2	35.4	39.4	37.9	39.7	37.5	0.1
Gly	8.5	7.8	7.9	7.4	8.7	8.0	0.5
Pro	10.5	10.2	10.6	9.9	10.9	10.5	0.1
Ser	10.0	9.4	9.3	8.9	10.5	9.6	0.1
Tyr	6.0	5.6	5.9	5.7	6.4	5.9	0.6

^1^CSBM: conventional soybean meal, FSBMA: soybean meal fermented by *Aspergillus oryzae* GB-107, FSBMB: soybean meal fermented by *Bacillus subtilis* PP6, UVFSBMB: UV-sterilized soybean meal fermented by *Bacillus subtilis* PP6, PSBM: probiotics-supplemented soybean meal, and SPC: soy protein concentrate.

### Animal management, digesta sampling and chemical analyses

All pigs were housed in individual metabolic crates (0.93 × 1.53 m^2^) in a room of steady temperature (27°C), controlled with a heating lamp. During a 14-d recuperation period, all pigs were fed the commercial starter diet containing 230 g/kg CP and allowed *ad libitum* access to feed and water. The initial BW for Groups 1 and 2 were 7.62 ± 0.52 and 10.44 ± 1.79 kg, respectively. After the collection phase of Group 1, the pigs were allotted to 7 dietary treatments again by CRD and the same adaptation and collection process were repeated. Each collection period was carried out over 7 d, consisting of 5 d of adaptation phase and 2 d of collection phase. The experimental diets were supplied twice a day at 0700 and 1900 with *ad libitum* access to water according to the rate of 2.0 times of the maintenance requirement for ME (NRC, 1998) based on initial BW of pigs. The ileal digesta were collected during 12 h from 0800 to 2000 for 2 d and the detailed collection procedure followed Jorgensen et al. [8]. The sterilized plastic bag (200 ml) was fitted to ileal cannula with cable tie. The bag was checked every 30 min and removed immediately when it exceeded a half level. The collected samples from each pig were placed in separate bags and stored in deep freezer, at −60°C to prevent bacterial AA degradation. At the end of collection phase, all collected samples were thawed, pooled and lyophilized to make a solid form using a freeze-dryer, then ground to pass 2 mm screen with a Wiley mill. After grinding, ileal digesta were mixed again and sampled for analysis. Diets were analyzed for dry matter (DM) by oven drying at 105°C for 3 h (935.29; [9]), and CP by the Kjedahl method (984.13; [9]). Chromic oxide in ileal digesta and feed were determined according to Fenton and Fenton [10]. For determination of stable AA levels except sulfur containing AAs, the samples were hydrolyzed at 110°C for 24 h with 5 ml 6 N hydrochloric acid per 20 mg sample. In case of sulfur containing AAs, the performic acid was used as a reagent for oxidation at the same level with hydrochloric acid. After acid hydrolysis, the hydrolysates were analyzed by the Beckman 6300 AA Analyzer (Beckman Instruments Corp., Palo Alto, CA) using ninhydrin reagent and the hydrolysate program.

### Calculations and statistical analysis

Each pig was used as an experimental unit. The AID of AA was calculated with chromium contents in the diets and digesta by the indirect method. Ileal endogenous AA losses, induced by the N-free diet, were used for calculating SID. The calculations of AID and SID of AA were conducted according to Stein et al. [6] as shown below:

$$ \begin{array}{l} AID\ \left(\%\right) = \left[1 - \left(A{A}_{\mathrm{ie}}/\ A{A}_{\mathrm{d}}\right)\ x\ \left(C{r}_{\mathrm{d}}/\ C{r}_{\mathrm{ie}}\right)\right]\ x\ 100\hfill \\ {} BEAL\ \left(g/ kg\  of\ DMI\right) = A{A}_{\mathrm{nie}}x\ \left(C{r}_{\mathrm{nd}}/\ C{r}_{\mathrm{nie}}\right)\hfill \\ {}SID\ \left(\%\right) = AID + \left[\left( BEAL\ /\ A{A}_{\mathrm{d}}\right)\ x\ 100\right]\hfill \end{array} $$

where, AA_d_ and Cr_d_ = ratio of AA and Cr in the diet

$$ \begin{array}{l}A{A}_{\mathrm{ie}} and\ C{r}_{\mathrm{ie}} = ratio\  of\  AA\  and\ Cr\  in\  the\  ileal\  digesta\hfill \\ {}C{r}_{\mathrm{nd}} = ratio\  of\ Cr\  in\  the\ N- free\  diet\ \left(g/ kg\  of\ DM\right)\hfill \\ {}A{A}_{nie} and\ C{r}_{nie} = ratio\  of\  AA\  and\ Cr\  in\  the\  ileal\  digesta\  of\ N- free\  diet\ \left(g/ kg\  of\ DM\right)\hfill \\ {} BEAL = Basal\  endogenous\  AA\  losses\hfill \end{array} $$

Statistical analysis of all data were performed using analysis of variance (ANOVA) and means were compared using least significant difference multiple range tests by the GLM procedure of SAS (SAS Inst. Inc., Cary, NC). Differences were declared significant at P < 0.05 and highly significant at P < 0.01. Single degree of freedom contrasts were conducted between different bacterial species for FSBM, between FSBMB and UVFSBMB, and between FSBMB and PSBM.

## Results

Fermented soybean products contained higher dry matter (DM), CP and AA contents compared to CSBM. Soy protein concentrate (SPC) had the greatest contents of DM, CP and AA among the soybean products (Table 3). However, there was no considerable change in chemical composition by *Bacillus subtilis* supplementation (PSBM) and ultraviolet (UV) sterilization (UVFSBMB) compared with CSBM and FSBMB, respectively.

**Table 3:** Chemical composition of analyzed soybean products

**Ingredients** ^**1**^						
**Items**	**CSBM**	**FSBMA**	**FSBMB**	**UVFSBMB**	**PSBM**	**SPC**
**Chemical composition, g/kg**						
Dry matter	886.8	905.4	902.5	901.4	889.3	938.8
Crude protein	466.5	510.7	508.9	495.5	463.9	685.7
**Amino acid composition**						
Dietary indispensable amino acids, g/kg						
Arg	31.8	33.9	32.2	33.0	31.6	45.1
His	12.6	12.1	12.1	12.2	12.4	15.4
Ile	21.1	20.5	21.6	21.0	21.4	28.1
Leu	40.6	43.1	41.1	41.9	39.9	60.1
Lys	25.7	31.9	31.2	30.8	26.2	44.6
Met	8.9	8.2	10.6	10.2	8.6	14.1
Phe	23.5	25.2	24.6	24.4	24.1	33.4
Thr	18.6	20.8	21.1	21.5	19.1	25.1
Val	21.6	23.3	22.7	22.4	20.8	30.2
Dietary dispensable amino acids, g/kg						
Ala	22.0	25.1	23.1	23.3	21.4	32.1
Asp	51.1	53.8	50.4	51.2	51.0	69.8
Cys	7.6	8.2	9.2	8.8	8.2	11.2
Glu	91.7	102.0	104.4	102.6	94.1	135.4
Gly	20.4	20.8	21.1	21.3	20.2	27.4
Pro	24.5	29.1	29.2	28.8	23.8	36.6
Ser	23.1	26.3	24.6	23.9	22.8	32.4
Tyr	14.4	15.2	14.8	14.7	14.1	21.1

^1^CSBM: conventional soybean meal, FSBMA: soybean meal fermented by *Aspergillus oryzae* GB-107, FSBMB: soybean meal fermented by *Bacillus subtilis* PP6, UVFSBMB: UV-sterilized soybean meal fermented by *Bacillus subtilis* PP6, PSBM: probiotics-supplemented soybean meal, and SPC: soy protein concentrate.

The AID of CP was not different among dietary treatments. However, lower AIDs of Ile, Phe, Val, and Tyr, were observed in the CSBM diet compared with the FSBMB diet (P < 0.05, Table 4). Moreover, the CSBM diet showed lower AID of Phe compared with the SPC diet (P < 0.05). When the FSBMB was added to diets, AIDs of all AAs except Met, Cys, and Glu were increased compared with the FSBMA diet (P < 0.05), resulting in greater average AIDs of indispensable (P < 0.01) and dispensable (P < 0.05) AAs. There was no difference on the AIDs of AAs between the FSBMB and UVFSBMB diets. However, pigs fed the PSBM diet showed lower AIDs of Lys and Pro compared with those fed the FSBMB diet (P < 0.05).

**Table 4:** Effects of different types of soybean meal on apparent ileal digestibility coefficients of crude protein and amino acids in weanling pigs fed corn based diets

**Treatments** ^**1**^							**SEM** ^**2**^
**Items**	**CSBM**	**FSBMA**	**FSBMB**	**UVFSBMB**	**PSBM**	**SPC**	
Crude protein	0.900	0.913	0.929	0.913	0.914	0.914	0.007
**Amino acid digestibility**							
Dietary indispensable amino acids							
Arg*	0.916	0.928	0.941	0.931	0.933	0.930	0.002
His*	0.878	0.897	0.913	0.899	0.893	0.898	0.007
Ile*	0.876^b^	0.894^ab^	0.908^a^	0.896^ab^	0.893^ab^	0.898^ab^	0.004
Leu*	0.890	0.910	0.921	0.911	0.911	0.917	0.006
Lys*, ***	0.882	0.898	0.918	0.899	0.891	0.905	0.007
Met	0.938	0.933	0.957	0.950	0.942	0.948	0.008
Phe*	0.886^b^	0.906^ab^	0.920^a^	0.909^ab^	0.906^ab^	0.914^a^	0.003
Thr**	0.809	0.834	0.846	0.829	0.822	0.826	0.008
Val*	0.826^b^	0.857^ab^	0.867^a^	0.863^ab^	0.848^ab^	0.855^ab^	0.004
Average*	0.886	0.900	0.916	0.903	0.899	0.904	0.006
Dietary dispensable amino acids							
Ala**	0.843	0.860	0.879	0.864	0.851	0.865	0.007
Asp**	0.870	0.886	0.909	0.897	0.885	0.882	0.008
Cys	0.831	0.816	0.850	0.830	0.815	0.794	0.012
Glu	0.897	0.902	0.904	0.879	0.912	0.900	0.007
Gly**	0.811	0.829	0.869	0.829	0.829	0.818	0.012
Pro**, ***	0.868	0.875	0.903	0.883	0.872	0.874	0.007
Ser**	0.874	0.886	0.905	0.893	0.885	0.894	0.006
Tyr*	0.885^b^	0.891^ab^	0.918^a^	0.907^ab^	0.901^ab^	0.902^ab^	0.003
Average**	0.856	0.865	0.888	0.868	0.864	0.861	0.008

^a-b^Means with different superscripts in the same row significantly differ (P < 0.05).

^1^CSBM: conventional soybean meal, FSBMA: soybean meal fermented by *Aspergillus oryzae* GB-107, FSBMB: soybean meal fermented by *Bacillus subtilis* PP6, UVFSBMB: UV-sterilized soybean meal fermented by *Bacillus subtilis* PP6, PSBM: probiotics-supplemented soybean meal, and SPC: soy protein concentrate.

^2^Standard error of mean.

*FSBMA vs. FSBMB (P < 0.01).

**FSBMA vs. FSBMB (P < 0.05).

***FSBMB vs. PSBM (P < 0.05).

Dietary treatments of soybean products had no significant difference on the SID of CP but lower SID of Ile was detected in the CSBM diet compared with the FSBMB, UVFSBMB, and SPC diets (P < 0.05, Table 5). Additionally, the CSBM diet showed lower SIDs of Phe and Val compared with the other diets, except for the PSBM and lower SID of Tyr compared with the FSBMB and UVFSBMB diets (P < 0.05). In comparison of the SIDs between the FSBMB and FSBMA diets, similar results with AIDs were observed (P < 0.05), resulting in higher average SIDs of indispensable (P < 0.01) and dispensable AAs (P < 0.05). The PSBM diet had lower SIDs of Lys, Ala, Pro, Ser, and Tyr compared with the FSBMB diet (P < 0.05). However, the SIDs of AAs between the UVFSBMB and FSBMB diets did not differ.

**Table 5:** Effects of different types of soybean meal on standardized ileal digestibility coefficients of crude protein and amino acids in weanling pigs fed corn based diets

	**Treatments** ^**1**^						**SEM** ^**2**^
**Items**	**CSBM**	**FSBMA**	**FSBMB**	**UVFSBMB**	**PSBM**	**SPC**	
**Crude protein**	**0.935**	**0.946**	**0.962**	**0.948**	**0.945**	**0.947**	**0.007**
**Amino acid digestibility**							
Dietary indispensable amino acids							
Arg*	0.939	0.948	0.964	0.955	0.952	0.952	0.005
His**	0.920	0.935	0.953	0.940	0.930	0.937	0.007
Ile*	0.918^b^	0.935^ab^	0.952^a^	0.943^a^	0.934^ab^	0.942^a^	0.004
Leu*	0.930	0.947	0.959	0.951	0.947	0.955	0.006
Lys**, ***	0.928	0.943	0.962	0.949	0.934	0.945	0.007
Met	1.004	0.991	1.011	1.006	0.992	1.003	0.007
Phe*	0.918^b^	0.936^a^	0.950^a^	0.941^a^	0.935^ab^	0.945^a^	0.003
Thr**	0.845	0.868	0.882	0.866	0.853	0.861	0.008
Val*	0.877^b^	0.905^a^	0.916^a^	0.915^a^	0.895^ab^	0.904^a^	0.004
Average*	0.928	0.939	0.955	0.944	0.935	0.942	0.005
Dietary dispensable amino acids							
Ala**, ***	0.859	0.875	0.894	0.879	0.865	0.880	0.007
Asp**	0.896	0.908	0.933	0.922	0.907	0.906	0.008
Cys	0.855	0.841	0.874	0.853	0.839	0.819	0.012
Glu	0.921	0.925	0.926	0.902	0.934	0.922	0.007
Gly**	0.835	0.851	0.893	0.855	0.851	0.842	0.013
Pro**, ***	0.941	0.945	0.973	0.957	0.940	0.945	0.007
Ser**, ***	0.908	0.918	0.940	0.929	0.916	0.927	0.007
Tyr*, ***	0.931^c^	0.934^bc^	0.962^a^	0.952^ab^	0.942^bc^	0.946^abc^	0.003
Average**	0.888	0.895	0.919	0.900	0.893	0.892	0.009

^a-c^Means with different superscripts in the same row significantly differ (P < 0.05).

^1^CSBM: conventional soybean meal, FSBMA: soybean meal fermented by *Aspergillus oryzae* GB-107, FSBMB: soybean meal fermented by *Bacillus subtilis* PP6, UVFSBMB: UV-sterilized soybean meal fermented by *Bacillus subtilis* PP6, PSBM: probiotics-supplemented soybean meal, and SPC: soy protein concentrate.

^2^Standard error of mean.

^3^Basal endogenous losses were calculated by protein free diet (g/kg): Arg, 0.28; His, 0.19; Ile, 0.35; Leu, 0.62; Lys, 0.51; Met, 0.21; Phe, 0.28; Thr, 0.27; Val, 0.43; Ala, 0.14; Asp, 0.48; Cys, 0.08; Glu, 0.86; Gly, 0.19; Pro, 0.74; Ser, 0.32; Tyr, 0.26.

*FSBMA vs. FSBMB (P < 0.01).

**FSBMA vs. FSBMB (P < 0.05).

***FSBMB vs. PSBM (P < 0.0

## Discussion

The chemical composition of the CSBM used in this study was similar with that used in previous studies [11,12]. The highest CP and AA contents in SPC among soybean products agreed with Lenehan et al. [13]. SPC was made by removing a portion of the carbohydrates from dehulled and defatted soybeans, resulting in high protein and less fat contents compared with the CSBM. The higher CP and AA contents of the fermented soybean products (FSBMA and FSBMB) compared with those of CSBM in this study also agreed with previous studies [14,15]. Feng et al. [16] demonstrated that soybean product fermented by *Aspergillus oryzae* had higher DM and CP contents than CSBM. These differences may be attributed to the reduction of carbohydrate content by fermentation [2]. Cervantes-Pham and Stein [12] reported that several oligosaccharides such as sucrose, stachyose, and raffinose in the CSBM could be degraded by α-galactosidase produced from *Aspergillus oryzae* and *Bacillus subtilis*, resulting in the increase of other nutrient concentrations. These results could offer possible explanations for higher CP and DM contents in the FSBMA and FSBMB than the CSBM.

In the case of UVFSBMB, there was no change by UV sterilization on ileal AA digestibility. Even though UV-radiation could lead to structural changes of proteins and lipids [17,18]. SBM is known to have anti-radiation activity [19]. However, no change in chemical composition by the UV-radiation in this study indicated that the UV-radiation might not affect the nutrient availability of FSBMB.

Probiotics supplementation to the CSBM diet did not change the CP and AA compositions compared with CSBM. Cervantes-Pham and Stein [12] reported that the reduced contents of oligosaccharides and ANFs were observed in enzyme-treated SBM and FSBM. However, there was a pretreatment time to degrade substances after mixing of enzyme or probiotics in CSBM. In this study, there was no fixed pretreatment time for diet mixing, which resulted in no difference in chemical composition of CP and AAs between CSBM and PSBM.

The AIDs and SIDs of CP and AAs in soybean products were greater than previous studies at a range of 5% to 10% [20,11,12], which may be associated with the amounts of daily feed intake. Moter and Stein [21] reported that a reduction of daily feed allowance for pigs progressively increased the AID of all indispensable AA, except for Lys, Met and Thr and the SID of all AA, except for Arg, Trp, Asp, Pro, and Tyr. Diebold et al. [22] also reported that the lower AID of CP and AAs in pigs allowed higher feeding levels up to 60 g/kg of body weight (BW). In this study, the daily feed allowance for pigs was twice the maintenance ME requirement based on their initial BW. However, the other studies provided 2.5 to 3 times of maintenance ME requirement [20,11,12]. These differences could affect the different ranges of digestibility results.

In spite of these findings, the improvements of the AID and SID of AAs were observed in the FSBMB diet compared with the CSBM diet in this study. Generally, the large amounts of SBM have not been used in the diets of weaning pigs because of the ANFs such as trypsin inhibitors, oligosaccharides, antigenic factors and lectin [23]. However, Hong et al. [2] reported that fermentation of SBM increased the amounts of small peptide (<20 kDa) and eliminated trypsin inhibitors by the microbial AA synthesis and breakdown. Feng et al. [5] also reported that FSBM showed high activities of protease and trypsin in duodenum and jejunum of weaning pigs, resulting in improved protein digestibility. Therefore, this result indicates that fermentation of SBM has the potential to improve ileal CP and AA availability.

Regarding the different bacterial species for SBM fermentation, FSBMB (fermented by *Bacillus subtilis*) had higher AIDs and SIDs of most AAs than FSBMA (fermented by *Aspergillus oryzae*). There is limited information about the effect of different fermenting microbial strains on the SIDs of AAs. Teng et al. [24] reported that the reduction of trypsin inhibitors contents of the FSBM were greater by *Bacillus subtilis* (96%) than by *Aspergillus oryzae* (82%), and the amount of small peptide increased from 5% to 63% in the FSBM by *Bacillus subtilis* and from 5% to 35% in the FSBM by *Aspergillus oryzae*. The reduced trypsin inhibitor concentration in SBM can improve protein and AA digestion [25]. Additionally the superior activity of protease and amylase with *Bacillus subtilis* compared with those of *Aspergillus niger* was demonstrated when *Parkia biglobosa* was utilized as a fermented ingredient [26]. These results could be employed to explain the digestibility difference between the FSBMA and FSBMB. *Bacillus subtilis* may have more potential to improve SBM availability than *Aspergillus oryzae*.

In this study, the pigs fed the SPC diet showed higher AID of Phe and SIDs of Ile, Phe and Val compared with those fed the CSBM diet. This result agreed with the previous studies in which SPC was produced by the removal of soluble carbohydrates from SBM [27] and had higher AIDs of AAs in SPC compared with those in CSBM, resulting from reduced concentrations of ANFs [28].

The supplementation of *Bacillus subtilis* to CSBM (PSBM) showed slight improvements on the AIDs and SIDs of some AAs compared with the CSBM, but lower AIDs of Lys and Pro and SIDs of Lys, Ala, Pro, Ser, and Tyr compared with the FSBMB. The responses of supplementation of *Bacillus subtilis* on ileal AA digestibility of pigs are inconsistent [29,30]. In this study, pretreatment time after *Bacillus subtilis* supplementation was not provided. However, FSBM was supplemented to the diet after finishing the fermentation process. This difference in the pretreatment time might affect the AID and SID of AAs, resulting in little improvement of the ileal AA digestibilities in the PSBM diet.

The UV-sterilization of FSBMB did not affect the ileal AA digestibilities. There is no evidence to demonstrate this effect. However, in this study, there is no difference in AA composition between the FSBMB and UVFSBMB. The only difference is that the supplemented *Bacillus subtilis* was killed by UV-sterilization, which means that the pigs fed UVFSBMB diet could not consume probiotics alive. Therefore, consumption of microbial residues after the fermentation process had no beneficial effect on the ileal AA digestibility because it is likely that the nutrients for *Bacillus subtilis* in the feed were already spent in fermentation.

## Conclusion

Supplementation of FSBM products in the diet of weaning pigs could improve AIDs and SIDs of AAs, and fermentation with *Bacillus subtilis* had more potential to improve ileal AA digestibility when compared with *Aspergillus oryzae*. However, supplementation of *Bacillus subtilis* had no positive effect on ileal AA digestibility in weaning pigs. Further study is needed to explain the possible mechanisms of different responses on ileal AA digestibility from different types of fermenting microbes for SBM.
